# Application of Wearable Insole Sensors in In-Place Running: Estimating Lower Limb Load Using Machine Learning

**DOI:** 10.3390/bios15020083

**Published:** 2025-02-01

**Authors:** Shipan Lang, Jun Yang, Yong Zhang, Pei Li, Xin Gou, Yuanzhu Chen, Chunbao Li, Heng Zhang

**Affiliations:** 1 College of Computer and Information Science & College of Software, Southwest University, Chongqing 400715, China; langshipan@email.swu.edu.cn (S.L.);; 2 Chongqing Institute of Green and Intelligent Technology, Chinese Academy of Sciences, Chongqing 400714, China; jyang@cigit.ac.cn (J.Y.); alixiaopei@cqu.edu.cn (P.L.);; 3 Department of Orthopedics, The Chinese PLA General Hospital, Beijing 100039, China

**Keywords:** vertical ground reaction force, tibial bone force, machine learning, pressure insole

## Abstract

Musculoskeletal injuries induced by high-intensity and repetitive physical activities represent one of the primary health concerns in the fields of public fitness and sports. Musculoskeletal injuries, often resulting from unscientific training practices, are particularly prevalent, with the tibia being especially vulnerable to fatigue-related damage. Current tibial load monitoring methods rely mainly on laboratory equipment and wearable devices, but datasets combining both sources are limited due to experimental complexities and signal synchronization challenges. Moreover, wearable-based algorithms often fail to capture deep signal features, hindering early detection and prevention of tibial fatigue injuries. In this study, we simultaneously collected data from laboratory equipment and wearable insole sensors during in-place running by volunteers, creating a dataset named WearLab-Leg. Based on this dataset, we developed a machine learning model integrating Temporal Convolutional Network (TCN) and Transformer modules to estimate vertical ground reaction force (vGRF) and tibia bone force (TBF) using insole pressure signals. Our model’s architecture effectively combines the advantages of local deep feature extraction and global modeling, and further introduces the Weight-MSELoss function to improve peak prediction performance. As a result, the model achieved a normalized root mean square error (NRMSE) of 7.33% for vGRF prediction and 10.64% for TBF prediction. Our dataset and proposed model offer a convenient solution for biomechanical monitoring in athletes and patients, providing reliable data and technical support for early warnings of fatigue-induced injuries.

## 1. Introduction

Repetitive impact forces during running are a major cause of many common running-related injuries, particularly stress fractures [1,2]. Stress fractures sustained during physical activity account for 10% of all sports injuries, with approximately 90% of these fractures occurring in the lower limbs [3]. The high incidence of overuse injuries in running highlights the need to examine running biomechanics and identify specific characteristics potentially associated with stress fracture injuries [4]. Among these, the tibia, as the most frequently affected site for stress fractures [5], warrants focused monitoring to reduce the risk of sports-related injuries and enhance athletic performance.

Current methods for monitoring or estimating lower limb loading primarily fall into three categories: (1) Direct monitoring of tissue stress and strain can be achieved through implanted devices, such as sensors embedded in the tibial plateau near the knee joint [6] or in the Achilles tendon to record in vivo forces [7]. Additionally, strain gauges can be inserted into the medial side of the tibial shaft to measure strain directly [8]. (2) Indirect estimation through biomechanical models (such as musculoskeletal and finite element models) [9,10]. Sasimontonkul [11] developed a 2-D optimization model of the lower limb with 21 attached muscles to calculate joint reaction forces (JRF), muscle forces, and bone contact forces at the ankle; Khassetarash [12] used a finite element model of the tibia-fibula complex to analyze potential changes in tibial strain during fatigued downhill running. (3) Estimation based on wearable device data, potentially enhanced with machine learning techniques. Wearable devices used to estimate lower limb loading often incorporate IMUs (Inertial Measurement Units), pressure insoles, or a combination of both. Among these, tibial acceleration is a commonly used surrogate measure for tibial impact force by clinicians and researchers [13]. Sihyun Ryu [14] highlighted the significant influence of distal tibial accelerometers on predicting peak ground reaction force (GRF) and loading rate. Caleb D. Johnson [15] positioned an IMU device on the anteromedial aspect of the distal right tibia to monitor vertical and resultant tibial impacts, comparing the impacts during treadmill and overground running. When combined with machine learning approaches, these methods enable more accurate force estimation. For example, Ryo Eguchi [16] employed a probabilistic machine learning model based on Gaussian Process Regression and data augmentation to estimate vertical GRF using insole data, achieving an average error of 8% or less. Similarly, Maryam Hajizadeh [17] utilized pressure insole signals to develop a Long Short-Term Memory (LSTM) neural network for vertical GRF estimation. L.J. Elstub [18] integrated IMUs and pressure sensors into shoes and trained a LASSO (Least Absolute Shrinkage and Selection Operator) regression algorithm to compute peak tibial forces. Compared to lab-based estimates derived from motion capture and instrumented treadmills, their approach achieved an average error of 5.7% for peak tibial force estimation. Overall, implantable devices can accurately monitor tibial forces but are unsuitable for daily sports activities due to their invasive nature and associated risks. Biomechanical modeling offers high accuracy but relies heavily on complex instruments and specialized expertise, making it is more applicable to small-scale experimental studies. In contrast, wearable sensors and devices are increasingly favored in human–machine interaction and routine kinematic monitoring due to their superior performance and convenience [19,20,21]. However, most current studies are limited to estimating peak tibial loads or instantaneous tibial impacts, failing to achieve real-time, continuous monitoring of loading throughout the entire course of physical activity. As a result, the dynamic changes in tibial loading during movement are lost, hindering early identification and prevention of tibial fatigue injuries.

**Figure 1:**
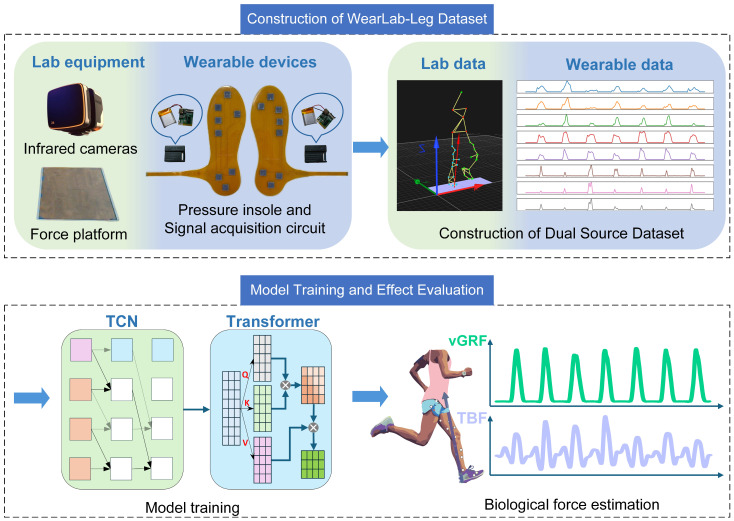
Flow chart of dataset construction and biological prediction. Among them, the waveform diagrams of different colors in Wearable data represent the motion data collected by the eight channel pressure insole. The different colors in the TCN and Transformer modules represent data features at different processing stages. TCN handles temporal dependencies, while Transformer utilizes Q, K, V for attention computation.

In this context, the contributions of this paper are illustrated in Figure 1. We recruited participants with no lower limb injuries to perform running exercises and simultaneously collected data from both a laboratory sports medicine platform (optical motion capture and force plates) and wearable insoles, constructing an Exercise-Based Lower Limb Dataset from Lab and Wearables (WearLab-Leg). Based on data from the laboratory equipment, we developed musculoskeletal and finite element models to further calculate muscle forces in various calf muscle groups and the distributed stress on the tibia during running. Additionally, our study combines Temporal Convolutional Network (TCN) and Transformer models to estimate the vertical ground reaction force (vGRF) and tibial bone force (TBF) from insole pressure data, enabling continuous monitoring throughout the entire running process. Our model combines the advantages of local feature extraction and global context modeling in biomechanical prediction. It leverages a multi-head attention mechanism to capture hidden dependencies in the signal sequence from different perspectives and further incorporates the Weight-MSELoss function to enhance peak prediction performance. Experimental results demonstrate that our model achieves superior performance in predicting TBF compared to traditional baseline models.

To facilitate further research and application, the code and dataset for this work are available on GitHub (at https://github.com/kytdhjrwe/BioForceNet, accessed on 14 November 2024).

## 2. Exercise-Based Lower Limb Dataset from Lab and Wearables (WearLab-Leg)

### 2.1. Dataset Construction

The Exercise-Based Lower Limb Dataset from Lab and Wearables (WearLab-Leg) was collected at the Sports Medicine Center of Southwest Hospital, comprising signals from both laboratory equipment and wearable devices. The laboratory’s Qualisys 3D motion capture and analysis system includes ten high-speed infrared cameras and two AMTI BMS400600 force plates, set to a sampling rate of 1200 Hz, allowing for synchronized collection of kinematic data and ground reaction forces (GRFs) from participants. The wearable insoles are custom-made eight-channel piezoresistive sensors with a sampling rate of 100 Hz, which replace the original insoles in participants’ athletic shoes to capture plantar pressure signals. Nine healthy volunteers, with no musculoskeletal injuries in the past six months and no chronic conditions affecting performance, were recruited for this study. Signals from both laboratory and wearable devices were synchronously collected during their exercise sessions. Of these volunteers, seven were male and two were female, aged 23.2 ± 1.78 years, with a height of 170.3 ± 12.3 cm and weight of 59.7 ± 9.7 kg. All participants provided written informed consent for the study and reviewed the “Volunteer Instructions” before the experiment.

**Figure 2:**
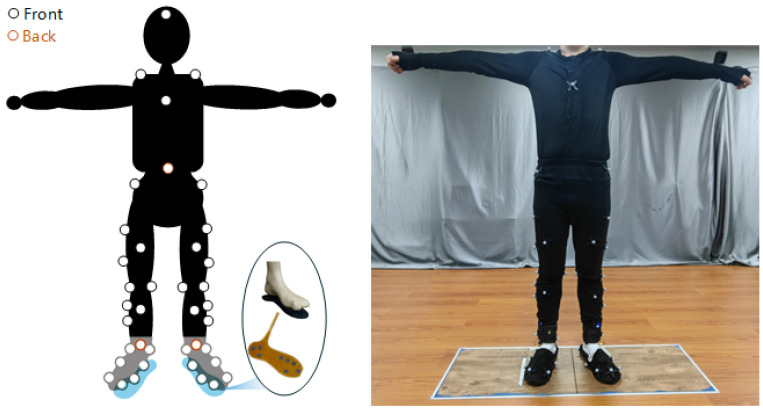
Marker point pasting position and wearing of insole equipment.

Before starting the experiment, calibration was performed following the manufacturer’s prescribed procedures. A global coordinate system was established as shown in “Lab data” in Figure 1, with the *X*-axis aligned with the longer edge of the first force platform, the *Y*-axis along the shorter edge, and the *Z*-axis pointing vertically upward, perpendicular to the X–Y plane. All captured marker positions on the subject and the ground reaction forces (GRFs) recorded by the force platforms were based on this coordinate system. The subjects then donned a tight motion capture suit and pressure insoles. A professional clinician identified anatomical bony landmarks to minimize adjustments during musculoskeletal model scaling. Subsequently, 39 reflective markers were attached to the subject as shown in Figure 2, following the gait2392 musculoskeletal model.

Once preparations were completed, the experiment officially began. The subject stood with arms outstretched and eyes forward on the two force platforms, maintaining a stationary position for 30 s. During this time, foot pressure data from the wearable insoles, optical motion capture data, and force platform data were synchronously collected. The optical motion capture system and force platforms were integrated into the same data acquisition system, eliminating the need for alignment. Data from the pressure insoles, however, were synchronized using timestamps. Each data point collected was assigned a timestamp, allowing high-sampling-rate motion capture and force platform data to be downsampled to match the sampling rate of the pressure insoles. This ensured all data were temporally aligned. Therefore, prior alignment of the pressure insoles with the motion capture coordinate system was unnecessary, as synchronization could be achieved post-collection. The purpose of collecting static data was as follows: the baseline data from the pressure insoles helped train machine learning models to recognize the initial pressure distribution across the insole’s channels; the static optical motion capture data enabled the construction of a static musculoskeletal model in OpenSim, aligning captured marker points with the model’s anatomical landmarks to correct errors from manual marker placement; and the force platform data provided the subject’s body weight, which was later used to normalize vertical GRFs and tibial forces. Next, an audio file with a moderate, steady “one-two, one-two” cue was played. Participants lifted each leg alternately to simulate running in place, following the cue and ensuring their feet fully contacted the force plates with each step. The experiment continued until participants were fatigued, after which all laboratory and wearable device data were saved, and the equipment was removed from the participants. The experiment was terminated early if any participant experienced discomfort during data collection or exercise.

### 2.2. Tibial Bone Force Calculation Using Laboratory Equipment

**Figure 3:**
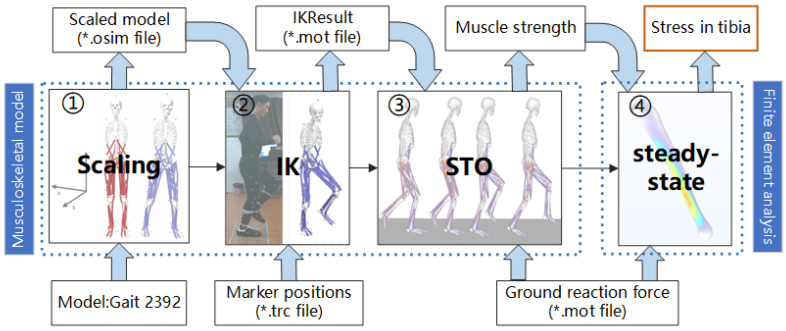
Flow chart of musculoskeletal model construction and tibial load calculation. Gait 2392 is a musculoskeletal model of the human body. The asterisk (*) denotes a filename, which can be customized during processing, but the file format remains fixed.

The process of calculating TBF using laboratory equipment requires the use of musculoskeletal modeling and finite element analysis. As shown in Figure 3, initial three steps consist of constructing a musculoskeletal model using OpenSim v.4.4 software, a freely available application and library for musculoskeletal modeling and simulation [22]. The musculoskeletal model takes as input the 3D trajectories of whole-body markers and GRFs and outputs the muscle forces in the surrounding tibial musculature. The final step, performed through COMSOL Multiphysics^®^ (COMSOL Multiphysics^®^ v. 6.0. cn.comsol.com. COMSOL AB, Stockholm, Sweden), involves constructing and analyzing the finite element model of the tibia. In this model, muscle forces and GRFs are inputted to calculate the Von Mises stress on the tibia [23].

The Qualisys 3D Motion Capture and Analysis System produces c3d files, which are subsequently processed with Matlab (MathWorks, R2022a, Natick, MA, USA) to separate the files into trc files containing trajectory information and mot files containing GRFs data for further analysis. In step ①, the original gait2392 model was scaled to match the subject by adjusting the model according to their height, weight, and fine-tuning the positions of key bony landmarks. Particular attention was given to landmarks that significantly influence lower limb load estimation, such as the medial and lateral malleoli, medial and lateral femoral condyles, and the tibial tuberosity. These points were meticulously aligned during scaling to minimize deviations and ensure accurate representation in the Scaled model.osim file. The trajectory data of the markers during movement is inputted into the scaled model in Step ② for Inverse Kinematics (IK) calculations, yielding joint displacements and rotation angles for the hip, knee, ankle, and other joints as IKResult.mot. In Step ③, Static Optimization (STO) decomposes the net joint moments at each time step into individual muscle forces based on joint positions, velocities, accelerations, and external forces (GRF), calculating muscle forces by minimizing the sum of squared muscle activations [24]. By analyzing the changes in muscle forces of various lower leg muscle groups throughout the gait cycle during running, the OpenSim simulation results were found to align with trends reported in other studies [25,26], as detailed in Appendix A, thereby validating the accuracy of the muscle force estimations. Anatomical references indicate that the lower leg comprises multiple muscles arranged from superficial to deep layers, each serving distinct functions. Some muscles have negligible effects on tibial loading. Thus, in subsequent finite element analysis (FEA) of the tibia, only the lateral gastrocnemius, medial gastrocnemius, soleus, posterior tibialis, and anterior tibialis muscles were included. Including all lower leg muscles in the analysis would not only increase complexity but also risk introducing data redundancy and computational resource limitations, potentially reducing efficiency and making the results harder to interpret and apply. The five selected muscles were chosen for their close anatomical connections to the tibia and their direct mechanical influence via tendons or muscle fibers. Their contributions to tibial loading are predominant, and many studies [27,28,29] have emphasized their critical roles in movement mechanics.

Next, a standard adult male tibia model was imported into COMSOL. This model, sourced from German manufacturer GOM, was created via CT scanning and calibration and is provided as a SolidWorks file. Based on [30], the tibia model parameters were set with a bone density of 1300 kg/m^3^, a Young’s modulus of 7 GPa, and a Poisson’s ratio of 0.3. Following the approach of Amir Hadid [31], a simple fixed constraint was applied to the distal end of the tibia, fully restricting translation and rotation at the tibia–talus interface. GRFs were applied as equivalent ankle joint forces at the contact area between the ankle joint and the tibia. Using coordinate data for the origins and insertions of major muscles as established in [32], the muscle force application points were identified on the model. In Step ④, muscle forces and GRFs were implemented frame by frame to the tibia model, calculating the Von Mises stress for each steady-state tibia segment, measured in N/m^2^. A stress distribution map was generated, clearly illustrating that maximum body stress occurs at the distal third of the tibia, as shown in Appendix B, aligning with the common occurrence of fatigue fractures in this region.

## 3. Estimation Model for Lower Limb Exercise Load Based on Insole Sensors

### 3.1. Problem Statement

Our objective is to monitor vGRF and TBF during running using pressure signals from wearable insoles, which presents a multivariate time-series forecasting problem. Let *X* represent the multivariate time series of a specific insole sensor, denoted as: *X* = [*x*_1_, *x*_2_, …, *x*_*T*_] where *T* = |*X*| signifies the length of *X*, and *x*_*i*_ ∈ *R*^*d*^ indicates a d-dimensional data point at time *i* ∈ [1, T] (the insole has an 8-channel sensor, hence d = 8). Let *Y* = [*y*_1_, *y*_2_, …, *y*_*T*_] represent the variations in vertical ground reaction forces over the time interval [1, T], and *Z* = [*z*_1_, *z*_2_, …, *z*_*T*_] denote the changes in tibial bone forces during the same interval [1, T], where both *y*_*i*_ and *z*_*i*_ are one-dimensional data points.

### 3.2. Model Architecture

**Figure 4:**
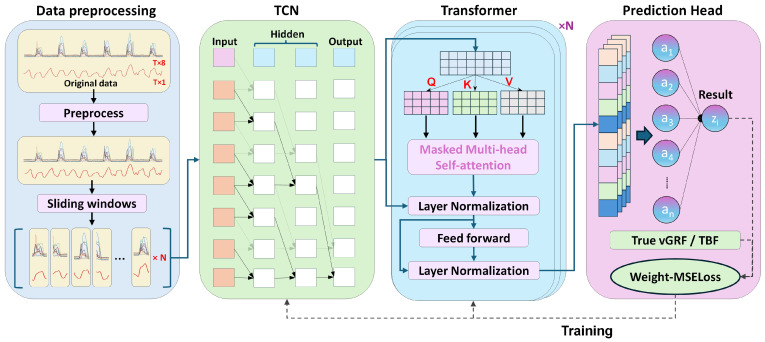
Estimation model for lower limb exercise load based on insole sensors. In the Data Preprocessing module, the upper section represents the raw data, which consists of eight-channel pressure insole signals and the target biomechanical forces to be estimated. After undergoing preprocessing steps such as filtering and normalization, the data is segmented into time windows of uniform length. The subsequent three modules are the TCN module, the Transformer module, and a prediction head, arranged sequentially.

The proposed model integrates a TCN and a Transformer Encoder to extract multi-scale features from time series and model complex temporal dependencies, ultimately achieving regression prediction through a prediction head, as illustrated in Figure 4. The first module preprocesses the collected data, including filtering, downsampling, and segmenting the data into time windows. After shuffling, the preprocessed data is loaded in batches using a DataLoader for model training. The second module is the TCN module, which extracts features from the input time series using a one-dimensional convolutional network architecture tailored for temporal data. The third module is the Transformer Encoder, which serves as the core encoding component of the model. By stacking multiple encoder blocks, it further processes the embedded features output from the TCN, thereby enriching temporal sequence modeling. The final module is the prediction head, which aggregates and normalizes the encoded features before passing them through a linear layer to generate the predicted vGRF or TBF.

### 3.3. TCN Module

To enhance the temporal modeling capability of the TCN module and capture longer dependencies, we implemented a stacked convolutional architecture [33]. Each convolutional layer employs varying dilation rates to expand the receptive field, ultimately mapping the features into an embedding space for subsequent use by the Transformer Encoder. The dilation rates for the first three convolutional layers are 1, 2, and 3, respectively, with each layer employing 40 convolutional filters of size 5. This setup gradually expands the receptive field to capture long-term dependencies. Following these, a convolutional layer with kernel size (4, 1) compresses the feature dimension, reducing the size of the temporal dimension. After convolution, BatchNorm2d [34] is applied to normalize the output, ensuring stable distribution across layers during training. The ELU activation function is then applied, followed by a pooling layer and a dropout layer with a rate of 0.5. Finally, a projection layer rearranges the multidimensional feature map into a flat embedding vector. The TCN module serves as a feature extractor within the model, utilizing dilated convolutions to gradually expand the receptive field, allowing the network to capture dependencies across different time scales within shallower layers without needing an excessively deep network structure.

### 3.4. Transformer Encoder

The Transformer Encoder module comprises multiple stacked Transformer Encoder Blocks, with each block containing a multi-head self-attention mechanism [35] and a Feed Forward Block. Residual connections and Layer Normalization allow the model to effectively capture complex long-term dependencies within time series data. The number of encoder blocks is determined by the depth parameter, which will be explored in detail later. Each encoder block independently processes the embedded features. For an input feature matrix X normalized by LayerNorm and denoted as $X\in R^{n\times \mathrm{embsize}}$, we set the emb size to 40. Next, three fully connected layers map the normalized features to three identical-shaped vectors: query (Q), key (K), and value (V), as shown in Equation (1).

(1) $$Q=XW_{Q},K=XW_{K},V=XW_{V}$$

In this context, $W_{Q},W_{K},{\mathrm{and}\;W}_{V}\in R^{\mathrm{em}\_\mathrm{size}\times \mathrm{emb}\_\mathrm{size}}$ represent learnable weight matrices. We compute the dot product between each query and all keys to obtain the attention scores, which reflect the similarity between each position in the query sequence and each position in the key sequence. To prevent large dot product values from causing numerical instability, the scores are scaled by dividing by the square root of the feature dimension, $\sqrt{d}$, and are subsequently normalized using softmax to yield the attention weights, as shown in Equation (2). During computation, multiple attention heads compute weights in parallel, with each head attending to distinct feature information [36].

(2) $$\mathrm{Attention}\left(Q,K\right)=\mathrm{softmax}\left(\frac{QK^{⊤}}{\sqrt{d}}\right)$$

Subsequently, the attention weights are employed to compute a weighted sum of the values, generating a weighted representation for each query position. Each value in the attention weight matrix indicates the degree of focus of the query position on each key position, with the elements of the value matrix being weighted accordingly to form a new output, as shown in Equation (3).

(3) $${\mathrm{output}}^{\left(h\right)}=\mathrm{Attention}\left(Q,K\right)\cdot V$$

Here, output represents the result of the weighted sum, representing the output of each attention head’s processing of the input; h denotes the attention head number, and we set a total of eight attention heads. Finally, the outputs from all attention heads are concatenated and linearly transformed by $W_{O}$, projecting them back to the original embedding dimension to yield the final multi-head attention output, as shown in Equation (4).

(4) $$\mathrm{MultiHead}\left(Q,K,V\right)=\mathrm{Concat}\left({\mathrm{output}}^{\left(1\right)},\ldots ,{\mathrm{output}}^{\left(\mathrm{num}\_\mathrm{heads}\right)}\right)W_{O}$$

In this setup, $W_{O}$ is a linear projection matrix, and the concatenated output has a shape of (batch_size, query_len, emb_size) consistent with the input embedding dimension. Here, batch_size denotes the size of the input batch and query_len represents the length of the query sequence.

### 3.5. Sliding Window Technique

The sliding window technique [37] is a method for processing data sequences by maintaining a fixed-size window that slides sequentially over the data, enabling the model to process segments of data rather than the entire dataset at once. We compared the biomechanical force estimation results after training the model using pressure insole signals divided into different time window sizes. A longer prediction window provides more information for biomechanical force estimation but may also introduce more noise or irrelevant details. In our experiments, a running gait cycle spans approximately 30 time-steps, and for the following discussions, the window size will be set to approximately one to three gait cycles in length.

### 3.6. Weighted Loss Function

In running, peak values of vGRF and TBF are of particular interest, as these peaks represent moments of high load, which are critical factors in fatigue accumulation and bone injury. Instead of using a traditional MSE loss function for training the multi-channel time series prediction model, we adopted a weighted loss function, referred to as Weight-MSELoss. By detecting the peaks of vGRF and TBF and assigning higher weights to these peak positions, the model’s focus on these critical peaks is enhanced, thereby improving the accuracy of peak fluctuation predictions. As shown in Equation (5), $y_{t}$ represents the true vGRF, ${\hat{y}}_{t}$ denotes the model’s predicted value, and the weight vector ${w=[w}_{1},w_{2},\ldots ,w_{T}]$ includes the adjusted weights for each time step t.

(5) $$\text{Weight-MSELoss}=\frac{1}{T}\overset{T}{\underset{t=1}{\sum }}w_{t}\cdot {\left(y_{t}-{\hat{y}}_{t}\right)}^{2}$$

After experimenting with different window widths, maxima in the vGRF with a minimum width greater than 12 were identified as peaks, and maxima in TBF with a minimum width greater than 8 were similarly identified as peaks. A base weight of 1 was assigned to all data points and the weights of the data points were increased within a radius of three time-steps around all peak positions. This is shown in Equation (6).

(6) $$w_{t}=\left\{\begin{array}{ll} \alpha (\alpha >1), & \mathrm{if}\;t\in \left[t_{peak}-3,t_{peak}+3\right]\\ 1, & \mathrm{Off}\;\mathrm{peak} \end{array}\right.$$

During the training process, the loss function optimizes the model parameters to minimize the loss value continuously. The traditional MSE loss treats the errors at all time points equally, leading to a gradient optimization process that is evenly distributed across the entire time series. However, the proposed weighted loss function shifts the optimization focus to key points (peaks) by increasing the value of $w_{t}$. Taking the gradient of the predicted value ${\hat{y}}_{t}$ in Equation (5), we obtain the expression in Equation (7).

(7) $$\frac{\partial (\text{Weight-MSELoss})}{\partial {\hat{y}}_{t}}=\frac{2}{T}\cdot w_{t}\cdot ({\hat{y}}_{t}-y_{t})$$

It can be observed that the gradient value is proportional to the weight $w_{t}$. In the peak regions where $w_{t}>1$, the gradient becomes larger, resulting in a faster error reduction rate in these regions. Consequently, the weighted loss function drives the model to prioritize minimizing errors at the peak points.

## 4. Experiments

### 4.1. Evaluation Metrics

The proposed model, a type of time series forecasting model, employs root mean squared error (RMSE), normalized RMSE (NRMSE), mean absolute error (MAE), R^2^ score (R2), and Pearson correlation coefficient as evaluation metrics. The RMSE is calculated as shown in Equation (8).

(8) $$RMSE=\sqrt{\frac{1}{n}\overset{n}{\underset{i=1}{\sum }}{\left(y_{i}-{\hat{y}}_{i}\right)}^{2}}$$

Since peaks in biomechanical force are of particular importance, we first compute the maximum peak value for each subject during running and then average of these values to normalize the RMSE, resulting in NRMSE, as shown in Equation (9). During running, vGRF approaches zero when the foot is off the ground, making MAE a more suitable metric than mean absolute percentage error (MAPE), as it avoids exaggerated error magnitudes that could hinder comparison, as shown in Equation (10).

(9) $$\mathrm{NRMSE}=\frac{RMSE}{\frac{1}{n}\sum_{i=1}^{n}\mathrm{Max}(y_{i})}$$

(10) $$MAE=\frac{1}{n}\overset{n}{\underset{i=1}{\sum }}\left|y_{i}-{\hat{y}}_{i}\right|$$

The R^2^ Score and Pearson Correlation Coefficient are used to assess the model’s fit and the correlation between predicted and actual values, with formulas shown in Equations (11) and (12), respectively.

(11) $$R^{2}=1-\frac{\sum {\left(y_{i}-{\hat{y}}_{i}\right)}^{2}}{\sum {\left(y_{i}-\bar{y}\right)}^{2}}$$

(12) $$\rho =\frac{\sum_{i=1}^{n}\left(x_{i}-\bar{x}\right)\left(y_{i}-\bar{y}\right)}{\sqrt{\sum_{i=1}^{n}{\left(x_{i}-\bar{x}\right)}^{2}}\sqrt{\sum_{i=1}^{n}{\left(y_{i}-\bar{y}\right)}^{2}}}$$

### 4.2. Baseline Methods

CNN and LSTM models have shown effectiveness in wearable device based human behavior and health monitoring applications [38,39,40]. For this study, we selected several baseline models capable of handling multivariate time series forecasting tasks: CNN, CNN-LSTM, CNN-BiLSTM, and BiLSTM-Attention, with MSE as the loss function. Hyperparameters were tuned through grid search, and the model architectures are detailed in Table 1.

- CNN: Extracts features from the time series through three convolutional layers and global average pooling, with final predictions made by a fully connected layer.
- CNN-LSTM: Features are first extracted by convolutional layers, and a unidirectional LSTM then captures long-term dependencies in the time series. The output is processed through a fully connected layer for prediction.
- CNN-BiLSTM: Local features are extracted via convolutional layers, followed by a bidirectional LSTM to capture forward and backward dependencies in the time series, with output through a fully connected layer.
- BiLSTM-Attention: A bidirectional LSTM captures temporal dependencies in both directions, with an attention mechanism highlighting important time-steps. The final output is produced by a fully connected layer.

### 4.3. Training Process

In the dataset construction section, vGRF can be directly obtained from force plates, while the overall Von Mises stress distribution of the tibia during running was derived using musculoskeletal modeling and finite element analysis. For each time step, the vGRF and the global maximum tibial stress are used as target values for estimation in this study. Prior to model training, preprocessing is required for the vGRF, TBF, and wearable insole data.

Since both vGRF and TBF are closely related to body weight, we normalized these forces based on each participant’s weight. For participant ${sub}_{p}$, the normalization formulas are shown in Equations (13) and (14).

(13) $$y_{norm}=\frac{y_{i}}{Weight_{sub\;p}}$$

(14) $$z_{norm}=\frac{z_{i}}{Weight_{sub\;p}}$$

A 15 Hz Butterworth low-pass filter was applied to the normalized biomechanical forces, and a 20 Hz Butterworth low-pass filter was applied to the insole sensor signals to reduce high-frequency noise and more accurately capture the main trends and features in the signals during movement. Each channel of the insole sensor signals was then normalized between 0 and 1 based on the maximum and minimum values, where $x_{n}$ represents the signal from the n-th channel, as shown in Equation (15).

(15) $$x_{n,norm}=\frac{x_{n}-\mathrm{Min}(x_{n})}{\mathrm{Max}(x_{n})-\mathrm{Min}(x_{n})}$$

The entire dataset was randomly shuffled and then divided into a training and test set with a 4:1 ratio. The training data was used to train the model, while the test data was used to evaluate model performance. During training, no test data was used, and hyperparameters were adjusted through grid search.

## 5. Results

This section presents the experimental results for predicting vGRF and TBF using the proposed model. We focus on the selection of sliding window size, encoder depth, the performance improvement from introducing a weighted loss function, and a comparison between the baseline models and our proposed model. All models were implemented using Tensorflow 2.16.1 and Python 3.10 and were trained on an RTX 3060 GPU with 3854 CUDA cores.

### 5.1. Effect of Window Size and Encoder Depth on Vertical Ground Reaction Force Estimation

To identify suitable model parameters for estimating vGRF, we compared prediction performance under different sliding window sizes and Transformer module depths. A gait cycle during running spans approximately 30 time-steps, so we explored window sizes ranging from one to three gait cycles by setting the window length to [30, 40, 50, 60, 70, 80]. The number of encoder layers was initially set to eight based on prior training experience. The resulting performance metrics are shown in Table 2.

With a sliding window size of either 50 or 60 time-steps, prediction performance was comparable, but the R2 score and correlation coefficient were higher with 60 time-steps; thus, the window size was fixed at 60 time-steps. Next, we investigated the impact of encoder depth on model performance. While the Transformer Encoder block serves to extract deep information, an increased number of blocks can add complexity, potentially degrading prediction performance. We varied the depth of the Transformer module between [1, 2, 3, 4, 5, 6, 7, 8], and the performance metrics are listed in Table 3.

**Figure 5:**
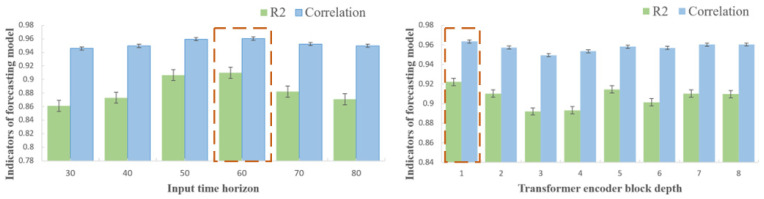
Performance of vGRF estimation model under different parameters. The red dotted frames represents the situation where optimal performance is achieved.

With an encoder depth of 1, the model achieved good prediction performance, but additional layers resulted in diminishing returns. However, as the encoder depth increased to five layers or more, performance was improved due to the extraction of richer information. Nonetheless, an encoder depth of 1 offered the best balance between model complexity and optimal performance. The R2 score and correlation coefficient are displayed in Figure 5, with results for different window sizes on the left and encoder depths on the right.

For the proposed model estimating vGRF, the optimal configuration was achieved with a window size of 60 time-steps and an encoder depth of 1. In this configuration, the R2 score was 0.9222, the Pearson correlation coefficient reached 0.9634, and the normalized root mean square error (NRMSE) was 7.77%.

### 5.2. Effect of Window Size and Transformer Depth on Tibial Bone Force Estimation

To identify optimal parameters for TBF prediction, we similarly evaluated model performance across different sliding window sizes and Transformer module depths. The TBF prediction during running is more complex than that for vGRF, so we extended the length of the time window to examine if longer sequences could help the model capture additional relevant information. Window lengths were set to [30, 40, 50, 60, 70, 80, 90, 100, 150, 200, 250], and the encoder depth was temporarily set to 8. The performance metrics are shown in Table 4. Due to the higher likelihood of prediction errors in TBF estimation, and the fact that R2 score and correlation coefficient may not fully capture subtle performance differences, we focused on MAE and NRMSE to compare model performance.

Performance was improved with increasing window size, but once the window length exceeded 80, NRMSE began to rise, and additional window length did not enhance model performance. Consequently, the window size for TBF prediction was set at 80 time-steps. Next, we varied the encoder depth from [1, 2, 3, 4, 5, 6, 7, 8], with performance metrics shown in Table 5.

**Figure 6:**
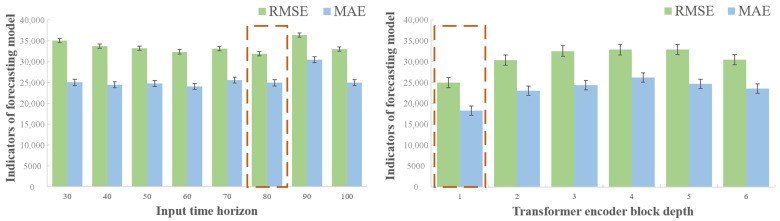
Performance of TBF estimation model under different parameters. The red dotted frames represents the situation where optimal performance is achieved.

With an encoder depth of 1, the model performed optimally, while additional encoders introduced unnecessary complexity, reducing generalizability. The model metrics are visualized in Figure 6, with results for different window sizes on the left and encoder depths on the right.

For estimating TBF using the proposed model, the optimal configuration was achieved with a window size of 80 time-steps and an encoder depth of 1, yielding a normalized root mean square error (NRMSE) of 12.05%.

### 5.3. Incorporation of a Weighted Loss Function

The weighted loss function was introduced to enhance the model’s ability to predict peak forces. The weights within the peak region are sequentially attempted to be set as [1, 2, 3, 4, 5, 6, 7, 8, 9], whereas the weights for all other data points, excluding those within the peak region, are set to 1. As shown in Table 6 and Table 7, increasing the peak weight does not always improve model performance, but certain weight configurations lead to significant enhancements. After introducing Weight-MSELoss, the NRMSE for vGRF prediction decreased by 0.44%, and for TBF prediction, it decreased by 1.41%.

**Figure 7:**
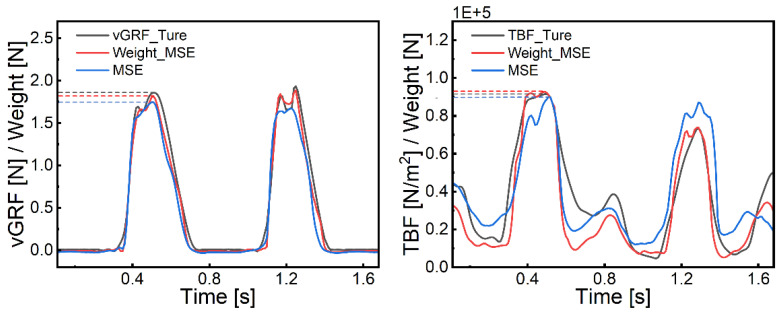
Improvement in peak prediction performance after introducing Weight-MSELoss.

Figure 7 illustrates the model’s peak force prediction after implementing Weight-MSELoss, where black represents true force, red represents predictions with Weight-MSELoss, and blue represents predictions using the traditional MSE loss function. The improvement in prediction performance is noticeable, particularly in peak force prediction accuracy.

### 5.4. Comparison of the Proposed Model with Baseline Models

Using grid search for parameter optimization, we trained each baseline model on the same dataset, and the resulting performance metrics are shown in Table 8. Compared to baseline models, for the simpler task of vGRF prediction, traditional models achieved NRMSE between 3.92% and 8.38%, with the best performance from the CNN + LSTM model. Our model achieved an NRMSE of 7.33%, within this range, but with the added benefit of improved peak force prediction due to its weighted focus on peaks.

For TBF prediction, traditional time series models struggled to extract deep information from the pressure insole data, leading to poorer performance. As shown in Table 9, baseline models achieved NRMSE between 11.83% and 15.51%, with the CNN model performing best. In contrast, our proposed TBF estimation model achieved an optimal NRMSE of 10.64%, outperforming the baseline models. Although current models are yet to precisely predict TBF during running, our model effectively captures the overall trend of TBF changes throughout the activity.

### 5.5. The Influence of Shoes on Lower Limb Load Estimation

To eliminate the influence of shoe material variability on plantar pressure measurements, all participants were provided with comfortable athletic shoes of uniform material and design. The original insoles were replaced with our custom insoles. Furthermore, to evaluate the extent to which shoes affect data collection for lower limb load calculations, an additional experiment was conducted with two participants performing in-place running on a force platform, both barefoot and wearing athletic shoes. Each participant selected their preferred comfortable running speed and performed in-place running while a metronome was used to ensure consistent step intervals. Ground reaction force (GRF) data were collected during the experiment. The collected data were segmented into gait cycles, and the metrics presented in Table 10 were calculated.

In the table, W represents the participant’s body weight in units of Newtons (N), and the values of $F_{f}$ and $F_{l}$ have been normalized by body weight. All results are presented as mean ± standard deviation, as shown in Table 11.

Observations revealed that the first peak and maximum vertical GRF values during barefoot running were higher than those during shod running. This difference is likely attributable to the cushioning effect of the shoes, which absorb a portion of the impact force. During barefoot running, cushioning relies entirely on active adjustments by muscles and joints, a process that requires time and results in a slight delay in the peak force. Conversely, the shoe sole’s cushioning properties during shod running absorb part of the impact, causing the first peak force to be reached more quickly. Additionally, the reduced contact area with the ground and shorter contact duration during barefoot running, combined with the absence of the shoe’s impact-dispersing effect, result in more rapid and pronounced GRF changes.

## 6. Discussion

### 6.1. Model Performance Analysis

With a gait cycle length of approximately 30 time-steps, a sliding window size of 60 produced optimal results for vGRF estimation, while a size of 80 was optimal for TBF estimation. Overall, data spanning two to three gait cycles contained comprehensive information. This window size selection not only enabled capture of key features in movement but also mitigated noise, enhancing model robustness and accuracy. For both vGRF and TBF estimation, the model achieved optimal performance with an encoder depth of 1, where additional encoder layers introduced unnecessary complexity. Furthermore, during training, deeper encoders led to overfitting, with better performance on the training set but poorer generalization on the test set. Thus, a shallow Transformer Encoder proved more suitable for predicting vGRF and TBF during running, achieving an NRMSE of 7.77% for vGRF and 12.05% for TBF. Finally, by replacing the loss function from traditional MSELoss to Weight-MSELoss, we further improved prediction accuracy and reduced estimation error, with an NRMSE reduction of 0.44% for vGRF and 1.41% for TBF.

### 6.2. Data and Model Interpretation

This study developed the WearLab-Leg dataset, a lower limb dataset for exercise-based analysis that synchronously collected data from a sports medicine platform and wearable devices (pressure insoles) during running. Using high-precision, real-time synchronized data, we detailed the process of constructing a musculoskeletal model and calculating global tibial Von Mises stress through finite element analysis. Based on this dataset, we proposed a model combining TCN and Transformer networks, which enables continuous monitoring of vGRF and TBF. For TBF prediction, the model incorporates a multi-head attention mechanism and a Weight-MSELoss function, which significantly enhances peak force prediction accuracy. The advantages of this model are as follows: (i) the multi-head attention mechanism facilitates the capture of hidden patterns and dependencies in the input sequence from multiple perspectives and levels; (ii) the combination of TCN and Transformer harnesses the benefits of local feature extraction and global context modeling for biomechanical force prediction; and (iii) Weight-MSELoss, compared to traditional loss functions, improves peak force prediction. In vGRF prediction, the model’s overall performance is on par with baseline models but with superior peak prediction. For TBF prediction, it captures deep information that traditional time series models fail to identify, outperforming baseline models overall.

### 6.3. Applications and Limitations

This study’s findings enable the prediction of vGRF and TBF throughout the entire exercise process, not just at peak forces, providing substantial benefits for future biomechanical monitoring of athletes or patients. This framework holds broad application, particularly for real-time monitoring of mechanical changes and potential risks during exercise. By predicting vGRF and TBF, it offers athletes personalized feedback to help adjust posture, optimize training loads, and reduce the risk of injury due to overtraining or improper technique. Additionally, this study demonstrates significant potential in the field of fatigue monitoring. As shown in Appendix C, the ability to perform long-term real-time monitoring of lower limb loading allows for the identification of early signs of fatigue accumulation and the analysis of individual fatigue responses under various training conditions.

However, this study has several limitations. To facilitate the capture of tibial forces transmitted from the plantar surface, the chosen activity was in-place running, which does not effectively reflect the actual lower limb loading during real running. This was an exploratory experiment using plantar pressure to estimate tibial forces. Future studies will extend experiments to instrumented treadmills to evaluate the effectiveness of the proposed method and model in real running scenarios. Another limitation lies in the finite element analysis. Using CT scans to create personalized tibial models for each participant, rather than relying on a generic finite element model, could further improve the accuracy of tibial force estimation. Additionally, accurately estimating tibial forces using only insole data remains challenging. The current approach primarily maps the upward force exerted on the tibia via the ankle joint and cannot capture the influence of lower leg muscle forces on the tibia during running. The development of portable and wearable devices capable of real-time muscle force monitoring in the future could enable more precise tibial force predictions, thereby enhancing the overall monitoring effectiveness.

## 7. Conclusions

Our study combines wearable technology and machine learning methods for data collection and load estimation of the lower limb during exercise. In response to the scarcity of datasets, this study constructs the WearLab-Leg dataset and outlines the complete process of musculoskeletal model construction and finite element analysis for tibial load calculation, providing a foundation for other researchers in lower limb load estimation. Based on this dataset, we propose a novel biomechanical force prediction framework to address the inability of existing human load estimation models to effectively extract deep information from wearable devices. This framework utilizes TCN networks with dilated convolutions to capture long-term dependencies, while a Transformer module with multi-head attention captures global dependencies. Additionally, the framework incorporates Weight-MSELoss, assigning higher weights to peak forces that contribute to bone fatigue accumulation and injury, thereby optimizing peak force prediction. Our study explores the impact of different window sizes on vGRF and TBF prediction, demonstrating that data spanning two to three gait cycles and a shallow Transformer Encoder are best suited for biomechanical force estimation. The model achieved an NRMSE of 7.77% for vGRF prediction and 12.05% for TBF prediction. With Weight-MSELoss, prediction accuracy improved further, achieving an NRMSE of 7.33% for vGRF and 10.64% for TBF. This framework meets the demands of routine biomechanical monitoring, providing reliable data support for exercise health management and fatigue injury early warning.

## Figures and Tables

**Table 1:** Hyperparameters of the baseline model.

	CNN	CNN-LSTM	CNN-BiLSTM	BiLSTM-Attention
Input shape	Time window size × 8	Time window size × 8	Time window size × 8	Time window size × 8
Layers 1	Conv1D filter size: 64; Kernel size: 3; Padding: 1; Activation function: ReLU	Conv1D filter size: 64; Kernel size: 3; Padding: 1; Activation function: ReLU	Conv1D filter size: 64; Kernel size: 3; Padding: 1; Activation function: ReLU	BiLSTM cells size: 64 (output: 64 × 2)
Layers 2	Conv1D filter size: 128; Kernel size: 3; Padding: 1; Activation function: ReLU	Conv1D filter size: 128; Kernel size: 3; Padding: 1; Activation function: ReLU	Conv1D filter size: 128; Kernel size: 3; Padding: 1; Activation function: ReLU	Attention block (Linear layer; Softmax: calculates attention scores; Splicing input and attention; Linear layer)
Layers 3	Conv1D filter size: 256; Kernel size: 3; Padding: 1; Activation function: ReLU	Conv1D filter size: 256; Kernel size: 3; Padding: 1; Activation function: ReLU	Conv1D filter size: 256; Kernel size: 3; Padding: 1; Activation function: ReLU	Flatten
Layers 4	GlobalAveragePooling1D	LSTM cells size: 64	BiLSTM cells size: 64 (output: 64 × 2)	Fully connected layer (output neurons: 1)
Layers 5	Fully connected layer (output neurons: 1)	Fully connected layer (output neurons: 1)	Fully connected layer (output neurons: 1)	

**Table 2:** Model estimation of vGRF performance under different sliding windows (The bold row in the table represents the case with the best performance).

Windows Size	R2	Correlation	MAE	RMSE	NRMSE
30	0.8610	0.9457	0.1361	0.2483	9.74%
40	0.8731	0.9494	0.1300	0.2369	9.29%
50	0.9066	0.9595	0.1143	0.2095	8.22%
**60**	**0.9097**	**0.9603**	**0.1126**	**0.2101**	**8.24%**
70	0.8822	0.9523	0.1233	0.2322	9.11%
80	0.8709	0.9498	0.1299	0.2386	9.36%

**Table 3:** Model estimation of vGRF performance for different encoder depths (The bold row in the table represents the case with the best performance).

Encoder Depth	R2	Correlation	MAE	RMSE	NRMSE
**1**	**0.9222**	**0.9634**	**0.1074**	**0.1982**	**7.77%**
2	0.9103	0.9572	0.1076	0.2123	8.33%
3	0.8920	0.9494	0.1169	0.2252	8.83%
4	0.8932	0.9535	0.1130	0.2214	8.68%
5	0.9146	0.9581	0.1078	0.2093	8.21%
6	0.9014	0.9569	0.1123	0.2176	8.53%
7	0.9102	0.9602	0.1095	0.2108	8.27%
8	0.9097	0.9603	0.1126	0.2101	8.24%

**Table 4:** Model estimation of TBF performance under different sliding windows (The bold row in the table represents the case with the best performance).

Windows Size	MAE	RMSE	NRMSE
30	25,033.934	35,082.727	16.94%
40	24,437.404	33,712.355	16.28%
50	24,793.752	33,184.844	16.02%
60	24,073.008	32,351.684	15.62%
70	25,612.310	33,122.940	15.99%
**80**	**24,932.357**	**31,899.58** **0**	**15.40%**
90	30,501.254	36,372.363	17.56%
100	24,996.023	33,031.830	15.95%
150	24,642.055	34,132.590	16.48%
200	26,310.150	33,327.910	16.09%
250	24,862.035	33,062.246	15.96%

**Table 5:** Model estimation of TBF performance for different encoder depths (The bold row in the table represents the case with the best performance).

Encoder Depth	MAE	RMSE	NRMSE
**1**	**18,263.76** **0**	**24,965.133**	**12.05%**
2	23,069.768	30,353.355	14.66%
3	24,328.816	32,531.336	15.71%
4	26,193.746	32,843.440	15.86%
5	24,639.256	32,861.004	15.87%
6	23,581.270	30,434.766	14.70%
7	25,095.387	34,444.637	16.63%
8	24,932.357	31,899.580	15.40%

**Table 6:** Estimation of vGRF in the Model after introducing Weight MSELoss (The bold row in the table represents the case with the best performance).

Different Weights	R2	Correlation	MAE	RMSE	NRMSE
MSELoss	0.9222	0.9634	0.1074	0.1982	7.77%
Weight2.0-MSELoss	0.9121	0.9618	0.1132	0.2055	8.06%
**Weight3.0-MSELoss**	**0.9302**	**0.9663**	**0.1004**	**0.1870**	**7.33%**
Weight4.0-MSELoss	0.9123	0.9594	0.1145	0.2085	8.18%

**Table 7:** Estimation of TBF in the Model after introducing Weight MSELoss (The bold row in the table represents the case with the best performance).

Different Weights	MAE	RMSE	NRMSE
MSELoss	18,263.760	24,965.133	12.05%
Weight2.0-MSELoss	17,378.994	24,051.031	11.61%
Weight3.0-MSELoss	16,801.295	23,283.740	11.24%
**Weight** **9.0-MSELoss**	**15,982.635**	**22,040.01** **0**	**10.64%**

**Table 8:** Comparison of vGRF prediction performance and baseline model (The bold row in the table represents the case with the best performance).

Model	R2	Correlation	MAE	RMSE	NRMSE
CNN	0.9148	0.9571	0.2137	0.1283	8.38%
**CNN + LSTM**	**0.9811**	**0.9907**	**0.0958**	**0.0551**	**3.76%**
CNN + BiLSTM	0.9794	0.9904	0.0999	0.0556	3.92%
BiLSTM + Attention	0.9552	0.9774	0.1518	0.0829	5.95%
Proposed model	0.9302	0.9663	0.1870	0.1004	7.33%

**Table 9:** Comparison of TBF prediction performance and baseline model (The bold row in the table represents the case with the best performance).

Model	MAE	RMSE	NRMSE
CNN	18,286.97	24,489.24	11.83%
CNN + LSTM	24,510.68	32,127.57	15.51%
CNN + BiLSTM	24,533.23	32,130.67	15.51%
BiLSTM + Attention	21,073.35	27,345.54	13.20%
**Proposed model**	**15,982.6** **4**	**22,040.01**	**10.64%**

**Table 10:** Definition and calculation method of dynamic indicators.

Metric	Definition	Calculation Formula	Unit
**First Peak Force**	The first peak value of the vertical ground reaction force during each gait cycle.	$F_{f}=\frac{F_{\mathrm{peak}}}{W}$	N/kg
**Time of First Peak**	The time corresponding to the first peak of the vertical ground reaction force during each cycle.	$T_{f}$	ms
**Maximum Force**	The maximum value of the vertical ground reaction force during each gait cycle.	$F_{l}=\frac{F_{\max}}{W}$	N/kg
**Contact Time**	The duration, within each gait cycle, from the moment the foot contacts the ground to its lift-off.	$\Delta_{t}=T_{off}-T_{touch}$	ms
**Maximum Loading Rate**	The rate of change in vertical ground reaction force within a unit time, measured as the steepest slope from the onset of force to the first peak.	$\begin{array}{c} K_{\max}=\max[\frac{F(n+1)-F(n)}{\Delta_{t}}]\\ \left(n=0,1,2,\ldots \ldots \right) \end{array}$	N/s

**Table 11:** Calculation results of dynamic indicators.

Metric	Subject 1		Subject 2	
	Barefoot	Wearing shoes	Barefoot	Wearing shoes
**First Peak Force (N/kg)**	2.15 ± 0.27	2.01 ± 0.23	1.56 ± 0.18	1.43 ± 0.16
**Time of First Peak (ms)**	92.96 ± 18.53	82.52 ± 18.90	92.86 ± 14.99	82.96 ± 13.19
**Maximum Force (N/kg)**	2.20 ± 0.21	2.13 ± 0.15	1.61 ± 0.14	1.56 ± 0.12
**Contact Time (ms)**	326.39 ± 26.41	336.80 ± 35.83	387.86 ± 64.16	436.24 ± 43.89
**Maximum Loading Rate (N/s)**	99,719.56 ± 50,564.22	75,369.79 ± 31,042.76	60,132.23 ± 28,641.63	52,561.34 ± 17,258.86

**Figure A1:**
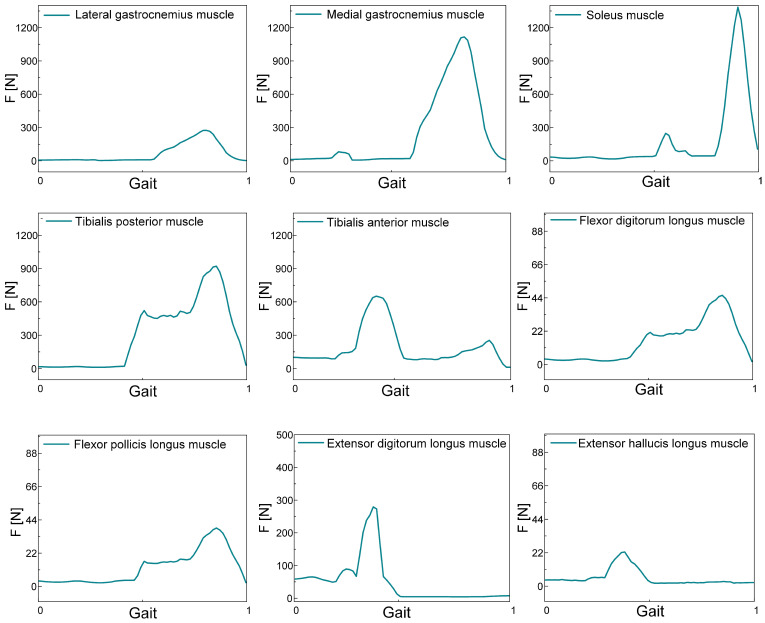
Variations in Muscle Forces of Calf Muscle Groups During a Gait Cycle.

**Figure A2:**
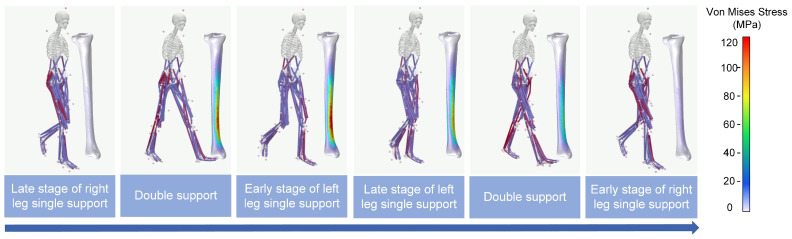
The tibial stress distribution in the left foot at various states throughout a complete gait cycle.

## Data Availability

The code and data that support the findings of this study are available on GitHub at https://github.com/kytdhjrwe/BioForceNet, accessed on 14 November 2024.

## Appendix A

In the second section, after constructing a musculoskeletal model using OpenSim, muscle forces of various muscle groups in the lower leg can be calculated through static optimization. The variation curves of these muscle forces within one gait cycle are illustrated in Figure A1.

## Appendix B

After finite element model calculations, the stress distribution variation of the left tibia over a complete gait cycle is shown in Figure A2. At the beginning of the gait cycle, the right leg is in a single support phase, while the left leg is fully suspended in the early swing phase. During this period, the main calf muscle groups exert minimal force, and there is no ground reaction force, resulting in low stress on the tibia. In the early double support phase, both legs are in contact with the ground, and the center of mass begins to shift from the right leg to the left leg. At this point, the left calf muscles start to activate, and under the combined effects of muscle force and ground reaction force, the tibial stress gradually increases. Next, in the left leg’s single support phase, the right leg is completely off the ground, and the body’s center of mass is fully transferred to the left leg. During this phase, the main calf muscles are fully engaged, leading to maximum tibial loading. In the latter part of the left leg’s single support phase, the right leg enters the swing phase and moves forward due to thigh muscle activity and inertia, causing a gradual decrease in tibial load. Once the right leg contacts the ground again, the body enters another double support phase, with the center of mass beginning to shift from the left leg to the right leg. During this transition, pelvic motion stabilizes, and the tibial load further decreases. Finally, as the right leg enters the support phase and the left leg transitions to the swing phase, the tibial load is reduced to a minimum.

## Appendix C

To demonstrate that the model can achieve long-term monitoring of tibial bone force (TBF), we conducted the following experiments: First, we input a 10-min segment of insole pressure data *X*_1_ into the model and compared the model’s predicted TBF with the TBF calculated from laboratory equipment to assess the error. Then, another 10-min segment of insole pressure data *X*_2_ was repeatedly input into the model 50 times, simulating a continuous 500-min running session in a real-world scenario. We evaluated whether the error between the model’s predicted TBF and the laboratory-calculated TBF changed across iterations. Finally, the original insole pressure data *X*_1_ was input into the model again to examine whether the model’s performance had degraded after long-term running. As shown in Table A1, the model’s predictive performance for TBF remained consistent throughout the running session. This consistency is attributed to the fact that the model’s parameters are fixed after training and are not updated during actual use, ensuring that performance does not degrade over time.

**Table A1:** Effects of Long term Running Simulation on Model Performance (The bold row in the table represents the case with the best performance).

Running Data Segment (10 min)	MAE	RMSE	NRMSE
***X*_1_**	**15,443.72**	**22,452.50**	**10.84%**
*X*_2_	20,965.49	26,777.84	12.93%
*X*_2_	20,965.49	26,777.84	12.93%
……	……	……	
*X*_2_	20,965.49	26,777.84	12.93%
***X*_1_**	**15,443.72**	**22,452.50**	**10.84%**

In addition, the insole sensor’s sampling rate is 100 Hz, corresponding to a sampling interval of 0.01 s. Using a 10-min dataset with 60,000 data segments as an example, we calculated the time required for the model to compute each TBF prediction and averaged the results. The average computation time was approximately 0.001 s, which is significantly less than the sampling interval of 0.01 s. This indicates that the model is capable of meeting the requirements for real-time monitoring.
